# Three-Dimensional Characterization of Potatoes Under Different Drying Methods: Quality Optimization for Hybrid Drying Approach

**DOI:** 10.3390/foods13223633

**Published:** 2024-11-14

**Authors:** Yinka Sikiru, Jitendra Paliwal, Chyngyz Erkinbaev

**Affiliations:** 1 Department of Biosystems Engineering, The University of Manitoba, E2-376, EITC, 75A Chancellor’s Circle, Winnipeg, MB R3T 5V6, Canada; 2 Research and Innovation Office, The University of Winnipeg, 515 Portage Avenue, Winnipeg, MB R3B 2E9, Canada

**Keywords:** potato drying, quality index, morphological features, energy consumption

## Abstract

The quality evaluation of processed potatoes is vital in the food industry. In this study, the effect of three different drying methods on the post-processing quality of potatoes utilizing 4, 8, 12, and 16 h of freeze drying (FD), infrared drying (ID), and oven drying (OD) was investigated. The impact of the drying methods on the potato’s microstructure was analyzed and quantified using 3D X-ray micro-computed tomography images. A new Hybrid Quality Score Evaluator (HQSE) was introduced and used to assess the Quality Index (QI) and Specific Energy Consumption Index (SECI) across various drying methods and durations. Mathematical models were developed to predict the optimal drying method. FD showed significantly higher (*p* < 0.05) colour retention, rehydration ratio, and total porosity, with minimal shrinkage, although it had higher energy consumption. ID had the shortest drying time, followed by OD and FD. The optimization showed that for FD, the optimal time of 5.78 h increased QI by 9.7% and SECI by 30.6%. The mathematical models could accurately predict the QI and SECI under different drying methods, balancing quality preservation with energy efficiency. The findings suggest that a hybrid drying system could optimize potato quality and energy consumption.

## 1. Introduction

Potatoes (*Solanum tuberosum* L.) are a globally cherished and economically significant crop, serving as a staple food for the growing world population with immense industrial applications. According to the Food and Agricultural Organization (FAO), approximately 375 million tonnes of potatoes were produced globally in 2023 [1]. The importance of potatoes is underpinned by their wide range of culinary applications, making them a fundamental ingredient in numerous dishes. However, proper preservation and processing techniques, such as drying, cold storage, and canning, are essential to harness their full potential and ensure their availability beyond harvest seasons [2]. Among several potato processing techniques, “drying” is one of the widely used industrial unit operations that significantly affect the quality characteristics of dried potatoes, such as texture, colour, flavour, and nutritional value. Therefore, finding the optimal drying conditions to produce high-quality dried potatoes with the best nutritional and functional properties is essential.

According to several research studies, it was evidenced that different drying methods affect the microstructure and morphology of potatoes [3]. Some of the commonly used methods are freeze drying (FD), oven drying (OD), infrared drying (ID), microwave drying (MD), and electrohydrodynamic drying (ED) [4,5,6]. These methods have different ways of transferring heat and mass, as well as varying levels of energy efficiency, duration, and product quality [6]. OD is a conventional method that uses hot air to remove moisture from the potato’s surface and interior. It is relatively simple and economical but can cause severe shrinkage, cracking, and hardening of the potato tissues [7]. FD uses a sublimation process for moisture removal and shows minimal sample shrinkage and damage to the potato tissue. However, it is energy-intensive, time-consuming, and expensive [4,7]. ID reduces drying time and energy consumption and improves the colour and rehydration of potato products by using electromagnetic radiation to heat the tissue directly and uniformly. However, this approach has limited penetration depth in the food material [8,9].

Several studies have been conducted on the effects of different drying methods on the quality and physico-chemical characteristics of dried potatoes [3,4,10]. Buzera et al. [11] studied the impact of different pre-treatment approaches and drying methods on the quality of potato flour. They found that FD results in lighter flour with good flow properties, while boiling and blanching at 95 °C using OD damages the starch granules and affects the flour quality. Similarly, Ando and Nei [4], revealed that potato samples subjected to microwave-vacuum drying tend to have higher porosity, while air-dried potato samples show significant shrinkage. In contrast to this, Wang et al. [3], compared air-impingement drying, infrared-assisted hot-air drying, and the hot-air drying of potatoes with temperature and humidity control. Their study showed that hot-air drying was the most efficient, offering faster drying, better colour retention, high ascorbic acid content, improved rehydration rate, and the least energy consumption rate.

The quality of dried potatoes can be determined by various imaging techniques. One of them is X-ray *μ*-CT, a non-destructive technique that can allow high-resolution 3D imaging of internal structures of various materials [12]. Prawiranto et al. [13] used the X-ray *μ*-CT to investigate the impact of different drying methods on the microstructure of apple fruit. The study showed that the various drying scenarios affected porosity, volume, shrinkage, connectivity, and pore diameter. Similarly, this 3D imaging technique was used to predict the microstructural changes in potato French Fries [14]. However, previous studies often focus on individual drying methods and their impact on specific individual quality. Despite the known benefits of hybrid drying systems that combine various methods such as FD, ID and OD, to optimize drying processes, there remains a significant gap in comprehensive studies that effectively evaluate their performance across multiple quality indices [5]. This fragmented approach necessitates a standardized metric that can integrate multiple quality indices to facilitate objective and quantifiable comparison. Moreover, existing established mathematical models such as Page models, the Lewis model, and others for predicting moisture ratio during drying fall short in predicting quality parameters such as rehydration ratio, texture, and others, an area of growing importance as consumer expectations for higher quality continue to rise [3]. These gaps limit our ability to fully optimize the drying process, particularly when it comes to product quality and energy efficiency.

To address these gaps, this study introduces the Hybrid Quality Score Evaluator (HQSE) to better understand the effect of different drying methods on potato quality. This innovative tool utilizes advanced 3D X-ray *μ*-CT to quantify microstructural changes and their impacts on quality and energy consumption. By integrating HQSE, new indices such as the Quality Index (QI) and the Specific Energy Consumption Index (SECI) were derived to develop mathematical models. These models aim to simultaneously predict optimal drying conditions that ensure high-quality preservation with minimal energy consumption. Therefore, this study investigated the three-dimensional microstructure transformations of dried potatoes through three different drying methods (FD, ID, and OD). Important morphological features extracted from the 3D hypercube developed by X-ray *μ*-CT were correlated to the postprocessing quality of potatoes. The HQSE was developed and used to assess the QI and SECI across various drying methods and durations. These introduced indexes allowed for the prediction of the optimal drying method using multiple objective optimizations, providing potential for hybrid drying techniques.

## 2. Materials and Methods

### 2.1. Sample Preparation

Potatoes (*S. tuberosum*) of the ‘Russet’ variety were obtained from a farm in Winnipeg, Canada, and stored at 4 °C before usage. All potatoes were appropriately washed and free from both physical and biological damage. The initial moisture content of the potato samples was measured using the air-oven drying method prior to analysis. Each potato sample was cut into a cylindrical form, with a diameter of 15 mm and a height of 15 mm, with a stainless-steel hollow pipe of 15 mm × 15 mm. Prior to the drying operation, all potato samples were subjected to blanching at 90 °C for 3 min [4]. Samples for the FD were subsequently frozen in a freezer (Model-IUE 40086FA; Thermo Fisher Scientific LLC, Waltham, MA, USA) at −80 °C.

### 2.2. Drying Methods

A Thermo Precision oven dryer (Model-6555; Thermoelectric Corporation, Chicago, IL, USA) was used for oven drying. Potato samples were placed inside the oven dryer at a temperature of 60 °C, and the timer was initiated at the commencement of the drying process. The samples were grouped into three sets for each drying duration (4, 8, 12, and 16 h). At the end of each drying duration for all drying methods, the trays were removed, and the weight of each sample was recorded.

Infrared drying was conducted using an infrared moisture analyzer (S/N-P1036951; A & B Company Ltd., Tokyo, Japan). Each set of the three samples was placed on a separate aluminum foil container, ensuring even spacing for efficient exposure to the infrared radiation, and labelled appropriately. The programme was set at a temperature of 60 °C and dried for three different periods 4, 8, 12 and 16 h.

Freeze drying was performed by a freeze dryer (CN-7934020; Labconco Corporation, Kansas City, MO, USA). The vacuum level was maintained at 0.110 mBar, and the collector temperature was set at –85 °C to facilitate the sublimation of frozen samples. The frozen potato samples were placed in a stainless-steel tray for drying, each covered with perforated aluminum foil. The samples were grouped into three sets for each drying duration (4, 8, 12, and 16 h).

### 2.3. X-Ray Micro-Computed Image Acquisition and Processing

**Figure 1:**
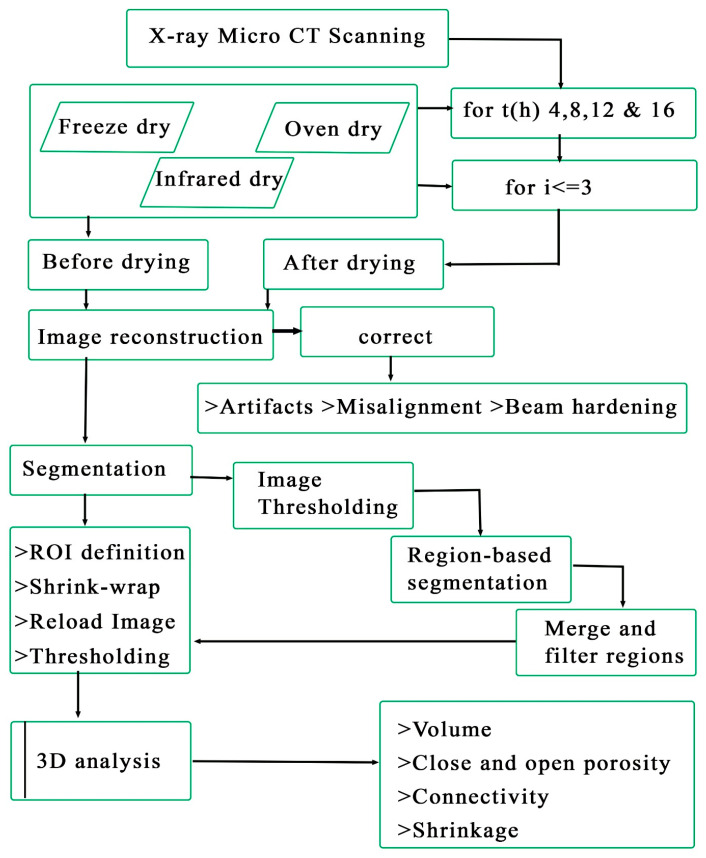
Flow chart of the image processing and analysis.

The three-dimensional structure of the potato samples, both pre and post-drying, was examined using X-ray *μ*-CT (model Skyscan 1275 by Bruker, Belgium). The image processing protocol is given in Figure 1. The system was set up for high-definition imaging (resolution of 1761 × 1382 pixels) under the following specific parameters: voltage at 40 kV, current at 180 μA, and rotational steps of 0.2° across a 180° arc [15]. This configuration generated a total of 1072 projections per sample. Potato samples were vertically mounted on a cylindrical metal rod using transparent wax to avoid vibrations during sample rotation while scanning. Altogether, 144 scans comprising three replicates per treatment were obtained before and after drying.

Bruker’s CTAn software (version 1.15) was used for quantifying the morphological features (total porosity, open porosity, closed porosity, object volume, and pore connectivity) of these samples. NRecon software (version 2.0, Bruker, Belgium) was used to reconstruct the high-resolution 2D images of the potato samples into a 3D hypercube stack.

### 2.4. Physical Parameters

#### 2.4.1. Porosity and Density Change

Total porosity (closed and open pores) is the proportion of air space/void space to the whole object area, and it was measured using the CTAn software from the reconstructed images. The three-dimensional images of the potato sample provided the morphological features that were used to estimate their density, such as the total volume [15]. Each potato sample was weighed before and after drying in three trials.

#### 2.4.2. Shrinkage Ratio

The shrinkage ratio (*SR*) of the potato samples after drying was expressed in percentage as area shrinkage ratio (*ASR*) and volume shrinkage ratio (*VSR*) following the method described by [5] with some modifications. Before and after drying, the potato samples’ dimensions (diameter and height) were measured from the X-ray images to determine the total integrated area and volume. The shrinkage ratio was calculated according to the equation below:

(1) $$ASR=\frac{\left(A_{B}-A_{A}\right)}{A_{B}}\times 100\%$$

where $A_{B}$ and $A_{A\;}$ represent the total surface area before and after drying, respectively.


(2) $$VSR=\;\frac{\left(V_{B}-V_{A}\right)}{V_{B}}\times 100\%$$

$V_{B}$ and $V_{A}$ represent the total volume before and after drying, respectively.

#### 2.4.3. Colour Measurement

The colour of the dried potato samples was determined using a Konica Minolta Chroma Meter (Model-CR-410; Minolta Corporation, Tokyo, Japan). All 144 samples were placed on a white ceramic tile, and the readings were taken as triplicates. The instrument was calibrated prior to analysis and after every 20 samples. The readings were directly compared with the respective readings of all fresh samples before subjecting them to drying. The colour change (ΔE) was calculated using the equation below [3].

(3) $$\Delta E=\sqrt{{\left(L_{0}^{*}-L^{*}\right)}^{2}+{\left(a_{0}^{*}-a^{*}\right)}^{2}+{\left(b_{0}^{*}-b^{*}\right)}^{2}}$$

where $L_{0}^{*}$ = the level of brightness (at 100) and darkness (at 0) before drying; $L^{*}$ = the colour output of the potato sample after drying; $a_{0}^{*}$ = the level of redness and greenness before drying; $a^{*}$ = the level of redness and greenness after drying; $b_{0}^{*}$ = the level of yellowness (+) and blueness (–) before drying; $b^{*}$ = the level of yellowness (+) and blueness (–) after drying.

#### 2.4.4. Rehydration Ratio

The rehydration ratio (RR) of the potato samples after drying was evaluated according to the method described by [5]. The weight of all 144 samples was recorded before and after rehydration, and the rehydration ratio was calculated as follows:

(4) $$RR=\frac{Mf}{Mi}$$

where *RR*—rehydration ratio in ${gg}^{-1}$; *Mf*—mass of potato sample after rehydration in g; *Mi*—mass of potato before rehydration in g

### 2.5. Drying Characteristics Analysis

#### 2.5.1. Moisture Ratio

The moisture ratio (MR) after drying for the three drying methods was calculated according to the given Equation (5) [2].

(5) $$M=\;\frac{M_{I+t}}{M_{I}}$$

where $M_{I}$ is the moisture content of the potato sample before drying on a dry basis (${gg}^{-1}$); $M_{I+t}$ is the moisture content of the potato sample after drying at t time on a dry basis (${gg}^{-1}$).

#### 2.5.2. The Drying Rate (DR)

The drying rate (*DR*) after drying for the three drying methods was calculated according to the given Equation (6) [2].

(6) $$\mathrm{DR}=\frac{M_{I+t}-{\;M}_{I}}{T_{I+4\;\;}-T_{I}}$$

where $M_{I+t}\;$ and $M_{I}$ are the moisture contents of potato samples at the drying times of $T_{I+4\;}$ and $T_{I}$ respectively, on a dry basis (${gg}^{-1}$); $T_{I}$ and $T_{I+4\;}$ are the initial and final drying times in h, respectively.

#### 2.5.3. Texture Analysis

The texture characteristics of the potato sample were expressed as hardness before and after drying using a Universal Test Frame LCI 200 load cell (Biosystems Engineering Laboratory, Winnipeg, MB, Canada). Readings were collected from 34972A data acquisition systems coupled with the instrument. A compression test was conducted using 889.6 N at a 52.4 mm spring to generate a plot of force (N) vs. voltage (V). This plot was used to develop the calibration curve for the analysis. For compression, all 144 samples were placed on a 120 mm by 60 mm rectangular metal plate. The test was carried out in a downward direction using a 15 mm diameter cylindrical steel probe, with a 10 mm travel distance at a speed of 2 mms^−1^. Hardness (N) was the average force at 10 mm penetration depth.

### 2.6. Specific Energy Consumption (SEC)

To obtain a more precise evaluation of energy consumption for each drying method (FD, ID, and OD), a detailed analysis of the specific energy consumption (SEC) was conducted. The SEC during the material drying process was calculated using Equation (7) [16].

(7) $$SEC=\frac{1000\;W}{Wi\;*\;MCi-Wf\;*\;MCf}$$

where *W* is the energy used during the experiment in kWh; *Wi* and *Wf* are the initial and final mass of the sample, in g; *MCi* and *MCf* are the moisture content before and after drying on wet basis (%).

### 2.7. Model Development

#### 2.7.1. The Hybrid Quality Score Evaluator

The HQSE was developed in MATLAB (version R2023a) to simulate and evaluate the performance of three drying methods: FD, ID, and OD. This algorithm integrates the quality index (QI) and specific energy consumption index (SECI) to provide an integrated evaluation of each method’s product quality and energy efficiency.

The HQSE consists of an input layer (drying time, physical and microstructural properties, SEC values) and a processing layer that handles the normalization and computation of QI and SECI, along with an output layer that combines these factors into a composite score. This structure was designed to sequentially process the inputs and derive an integrated output reflecting quality and energy considerations. The HQSE provides a singular score reflective of multiple quality factors, including colour, hardness, total porosity, shrinkage ratio, moisture ratio, and drying rate. Each parameter is weighted by its relative impact on the overall quality derived from normalized values, as shown in Equations (8)–(10).

#### 2.7.2. Data Normalization and Model Validation

Data were normalized between 0 (low) to 1 (high) index to ensure comparability [3]. Quality parameters where higher values indicated better quality (Group 1—rehydration ratio, drying rate, total porosity, hardness) were normalized directly, as shown in Equation (8). However, for those where lower values were preferable (Group 2—colour change, area and volume shrinkage ratio, moisture ratio, SECI), an inversion process was applied, as indicated in Equations (9) and (10). This aligns all indices to a common evaluation framework where higher indices uniformly represent better quality and energy efficiency performance, simplifying the assessment across all drying times and methods.

Utilizing the outputs from HQSE, two models were developed. The model selection was based on the highest coefficients of determination (*R*^2^) and lowest root mean square errors (RMSE), as reported in Table 1 The linear regression model was developed to explore the relationship between drying time and SECI. A polynomial model was also formulated to capture the more complex relationship between drying time and QI. A polynomial model was chosen based on the observed non-linear trends as quality changes over time. The models were validated with a data subset, using RMSE and *R*^2^ as the performance index. This validation process ensures the accuracy of predictions used to balance product quality with energy efficiency optimally.

The normalization equations of quality parameters are listed as follows:

For parameters where higher values are better (Group 1):

(8) $$QI=\frac{Qt-Qmin\;}{Qmax-Qmin\;}$$

For parameters where lower values are better (Group 2):

(9) $$QI=1\;-\;\frac{Qt-Qmin\;}{Qmax-Qmin\;}$$

*SEC* normalization (assuming lower *SEC* is better):

(10) $$SEC=1\;-\;\frac{SECt-SECmin\;}{SECmax-SECmin}$$

(11) $$\mathrm{Average}QI=\frac{1}{N}\sum_{k=1}^{N}QI$$

(12) $$CQI=\sum_{k=1}^{N}QI$$

where *N* is the number of quality parameters; *Qt* is the quality value at time *t*; *SECt* is the specific energy consumption at time t; *Qmin* is the minimum quality value across the drying time; *Qmax* is the maximum quality value across the drying time; *QI* is the quality index; *SECI* is the specific energy consumption index; *SECmin* is the minimum specific energy consumption across the drying time; *SECmax* is the maximum specific energy consumption across the drying time; *CQI* is the composite quality index.

#### 2.7.3. Multi-Objective Optimization Framework

Multi-objective optimization approach was incorporated to find the optimal drying conditions that balance high-quality preservation with minimal energy consumption. Using the HQSE-derived mathematical models, optimization models were constructed to perform multiple optimizations across various drying methods and durations. The optimization process employed the genetic algorithm (GA) to maximize QI and minimize SECI. The drying times for FD, ID, and OD were optimized to identify the best compromise between product quality and energy consumption.

#### 2.7.4. Statistical Analysis

The experimental data from the three drying methods and time were expressed as mean and standard deviation. All experiments were performed in triplicates. All data were analyzed as a one-way analysis of variance (ANOVA), and mean comparison was carried out using Tukey HSD at *p* < 0.05 confidence level using SPSS statistics (Version 22.0, SPSS Inc., Chicago, IL, USA).

## 3. Results and Discussion

### 3.1. Three-Dimensional Morphological Characterization

**Figure 2:**
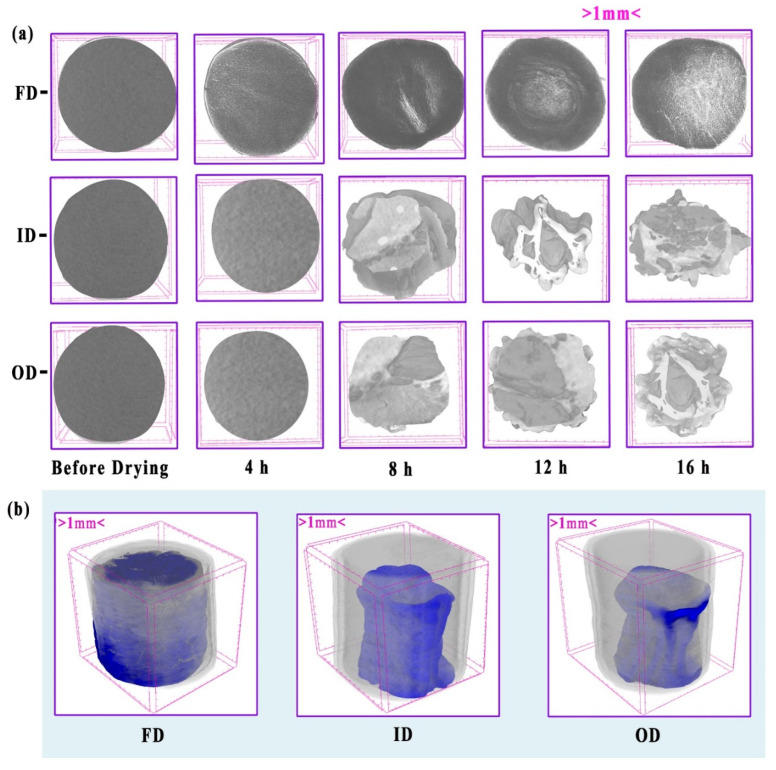
The morphological changes in potatoes post drying by freeze drying (FD), infrared drying (ID), and oven drying (OD): (**a**)—top view of 3D images of potatoes after 3 mm penetration, showing changes in pores after drying; (**b**)—isometric view of potato samples dried for 16 h with the transparency of the fresh sample reduced to 3% and the dried sample to 97%.

The effect of drying methods on microstructural changes in potatoes at different drying times was analyzed using 3D X-ray *μ*-CT imaging. The analysis included pore distribution, shape, and pore connectivity. A top view of the 3D images of potato samples before and after drying is presented in Figure 2a. A noticeable increase in porosity for all tested drying methods was observed with increased drying time. Merged 3D images of samples before and after drying are shown in Figure 2b. The transparency was adjusted to 3% before drying and 97% after drying to facilitate easier visualization. It was observed that the shrinkage ratio at 16 h of drying was significantly higher (*p* < 0.05) for both ID and OD samples, with a minimum shrinkage ratio observed for FD samples (Figure 2b).

#### Changes in Porosity and Density of the Dried Potatoes

**Figure 3:**
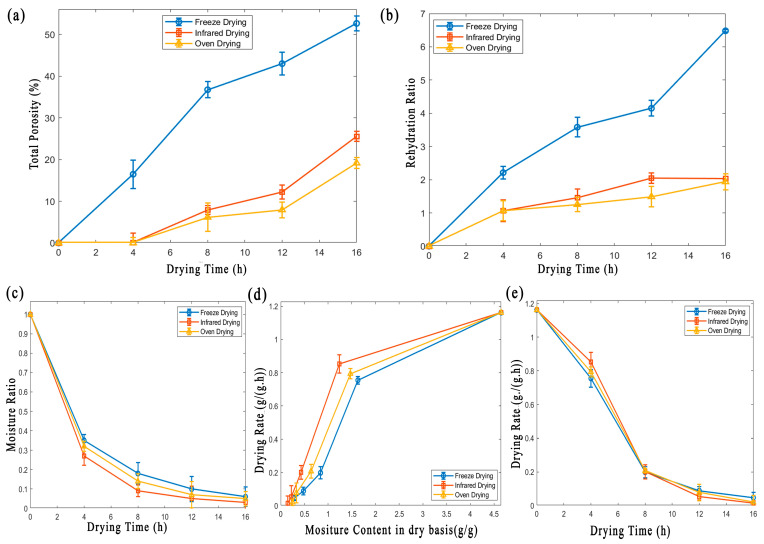
A comparative analysis of potato drying dynamics: (**a**) changes in total porosity with time across different drying methods; (**b**) the effect of different drying methods on the rehydration ratio of dried potato; (**c**–**e**) drying kinematics curves of potato under three drying methods.

The porosity of dehydrated potato slices is crucial for texture, rehydration, and overall product quality. This study quantified porosity changes in FD, ID, and OD samples at different drying times (Figure 3a, Table 2). Drying methods significantly affected porosity (*p* < 0.05). FD samples showed distinctive structural patterns. Total porosity increased significantly (*p* < 0.05) from 16.5% at 4 h to 52.69% at 16 h, predominantly due to the formation of open porosity [3,17]. This aligns with FD’s gentle drying process, which maintains interconnected voids without extensive closures. A similar finding was reported by Levin et al. [18] on the FD of coffee granules, where the freezing process parameters preserved internal structure and increased porosity [18]. In contrast, ID samples had low initial porosity (6.82 × 10^−4^% at 4 h) with no open porosity, reflecting the direct heat transfer of ID [9]. Total porosity increased to 25.5% at 16 h, with significant open and closed porosity development (Figure 2a). OD samples exhibited similar trends to ID, with minimal initial porosity (7.89 × 10^−4^% at 4 h) and an increase to 19.45% at 16 h, showing a more intricate porous network.

Density changes were also significant (*p* < 0.05) across drying methods (Table 3). FD samples showed negative density changes for all durations, showing the highest reduction of 72.09% at 16 h. This is because of continuous moisture removal and minimal shrinkage facilitated by FD [17,19]. The rapid evaporation in ID caused controlled shrinkage and moderated density change, with a minimum decrement of 6.58% at 4 h [8]. OD samples had significantly higher (*p* < 0.05) densities than FD or ID samples. OD caused considerable shrinkage, leading to an increase in density changes at 12 and 16 h. The highest increase in density was 12.54% at 12 h due to volume reduction and mass loss balance [9,19]. The fluctuating density in OD reflects the variation between volume shrinkage and mass reduction over time. This change in density impacts the sample’s quality by altering texture and structural integrity, which is crucial for transportation, storage, and various industrial applications [20].

### 3.2. Drying Characteristics

#### 3.2.1. Moisture Ratio

The moisture ratio is crucial in determining the water content in fruits and vegetables, affecting structural, sensory, textural, nutritional, and visual properties. The study showed a significant decrease (*p* < 0.05) in moisture ratio across all drying methods as drying time increased (Figure 3c). This reduction is due to continuous heat transfer, which accelerates moisture evaporation [21]. ID exhibited the lowest moisture ratio (*p* < 0.05) at all drying times compared to FD and OD. This highlights the efficiency of infrared in moisture removal due to rapid and uniform heat transmission [16]. In contrast, OD had a lower moisture ratio than FD, indicating more efficient moisture removal during drying (Figure 3c). The moisture ratio peaked at 4 h and gradually decreased at 8, 12, and 16 h across all methods. This pattern aligns with the findings by Sakare et al. [8], highlighting the effectiveness of infrared energy in accelerating the drying process and maintaining the product quality of food materials.

#### 3.2.2. Drying Rate

The drying rate of potato samples under FD, ID, and OD is shown in Figure 3d. ANOVA results indicated a significant (*p* < 0.05) effect of moisture content on the drying rate. The drying process occurred entirely during the falling rate period. This is consistent with a previous study by Wang et al. [3] during the drying of potato slices. Initially, the drying rate was high for all methods and decreased significantly (*p* < 0.05) as drying time increased due to the removal of tightly bound water molecules [22,23]. ID had the highest initial drying rate (*p* < 0.05) due to better heat penetration from infrared radiation. Still, this rate decreased as moisture content fell below 0.43 ${g(gh)}^{-1}$ g due to case hardening and minimum available moisture content [19]. In the late drying period, FD samples showed the fastest drying rate (*p* < 0.05) due to their higher moisture content and minimal shrinkage compared to ID and OD [24]. This facilitated more sublimation and increased surface area exposure to vacuum, enhancing the drying rate [10].

### 3.3. Hybrid Quality Score Evaluator (HQSE)

**Figure 4:**
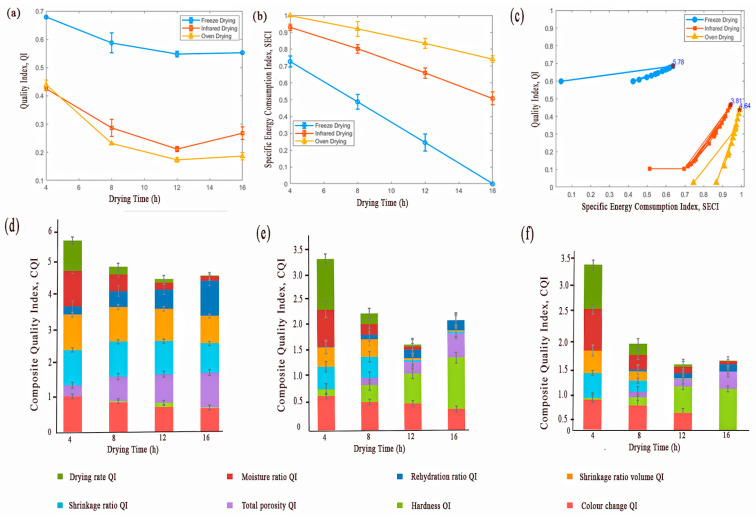
Quality and energy consumption analysis of potato drying processes: (**a**) changes in the quality index; (**b**) specific energy consumption index with time across different drying methods; (**c**) Pareto fronts of drying methods for optimizing quality and energy efficiency in potato drying; and a stacked bar graph illustrating the composite quality score for potatoes subjected to (**d**) freeze drying, (**e**) infrared drying, and (**f**) oven drying across various drying times. Each segment represents the weighted contribution of individual quality parameters—colour change, hardness, total porosity, area and volume shrinkage ratio, rehydration ratio, moisture ratio, and drying rate—to the composite quality index for each drying method.

The analysis of the QI for the three drying methods revealed distinct performance characteristics for each drying method (Figure 4). The results of various QI values, such as colour retention, hardness, total porosity, shrinkage ratio, rehydration ratio, moisture ratio, and drying rate, each contributing to the composite quality index (CQI), are illustrated in Figure 4. Higher total porosity QI values of 1.00 and 0.82 at 16 and 12 h, respectively, denote a desirable porous structure in FD samples that facilitates rehydration. This is crucial in ensuring process quality, as indicated by its significant contribution to the CQI. The shrinkage ratio, both in area and volume, contributes to the CQI, as it reflects the extent of physical alteration from the drying process. As shown in Figure 4, the higher volumetric shrinkage QI, 0.77 at 16 h in FD, highlights minimal dimensional changes in structure, thereby preserving the original shape and size of the potatoes [25]. The rehydration ratio, a direct consequence of porosity, emerges as a notable contributor to the CQI [18]. Higher rehydration ratio QI values in FD samples indicate minimal structural and cellular damage during drying, resulting in a superior ability to regain moisture. This characteristic is essential for products like ready-to-cook dehydrated potatoes and vegetables, which rely on maintaining texture and quality for reconstitution. Moisture ratio and drying rate QI provide insights into the drying kinetics and efficiency [26]. A higher moisture ratio QI (*p* < 0.05), as observed in ID (Figure 4e), is indicative of an efficient drying process essential for prolonging shelf life and preventing microbial growth. The faster drying rate reflects the process time efficiency, with a higher QI denoting cost-effective feasibility in industrial applications.

According to Figure 4, FD consistently showed a high QI across all drying times compared to the other two tested methods. However, a significant decline (*p* < 0.05) in QI was observed in later drying stages, possibly due to over-drying (Figure 4a). These findings suggest a critical need for precise control over drying parameters to optimize the quality [27]. In contrast, ID demonstrated an intermediate QI of 0.44 at 4 h, which suggests a better balance between efficiency and quality preservation of the dried potato samples (Figure 4a). The significantly lower (*p* < 0.05) moisture removal shown at the initial phases of ID may be attributed to the direct and uniform transmission of infrared energy to the potato tissues [26]. Therefore, it is evidenced that ID accelerates the drying process, enhancing early-stage product quality [28,29]. However, it was found that the rehydration ratio and total porosity were significantly decreased (*p* < 0.05) at the initial drying stages of ID. (Figure 4e). This suggests that prolonged exposure time might adversely affect certain quality aspects, such as rehydration capability and structural preservation, while ID is efficient in initial moisture removal (Figure 3e and Figure 4e). OD performance was similar to ID in terms of average QI at 4 h (Figure 4a), with an initial preservation of quality that gradually declined over drying time. The decline in the average QI for OD was more pronounced in colour change (0.38 ± 0.28) and rehydration ratio (0.07 ± 0.06) (Figure 4f), highlighting potential overheating issues and their detrimental effect on product quality [30]. In contrast to the QI results, the SECI analysis indicated that both ID and OD outperformed FD in terms of energy usage, with OD showing the most significant SECI (*p* < 0.05), particularly in prolonged drying processes (Figure 4b).

**Figure 5:**
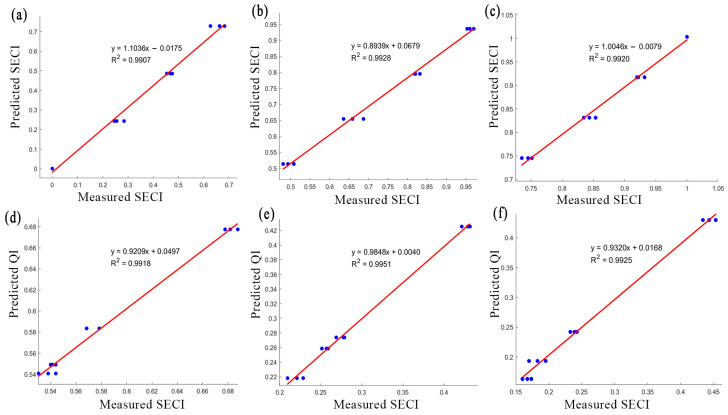
Comparative validation of predictive models against measured data showcasing (**a**) freeze drying; (**b**) infrared drying; (**c**) oven drying methods. Each subplot illustrates the correlation between the predicted quality index (**d**–**f**), QI, and specific energy consumption index, SECI, against their respective experimental outcomes.

The linear regression analysis revealed a significant relationship between drying time and SECI for the three tested drying methods. As shown in Table 1 all three derived models indicate a gradual decrease (*p* < 0.05) in SECI for all drying methods as the drying process progresses, and highlight a higher potential for energy optimization in drying processes. A polynomial model was formulated to assess QI for all drying methods (Table 1), highlighting a non-linear relationship between QI and drying time. It provides insights into quality deterioration and offers empirical evidence to guide the modification of the drying process to maintain product integrity and consistency. The validation using a subset of the data demonstrated that both models accurately predicted the SECI and QI (Figure 5). Plots comparing the predicted values against the actual experimental data revealed high accuracy with higher R^2^ and lower RSME (Table 1).

### 3.4. Multi-Objective Optimization

The optimization of drying times for FD, ID, and OD focused on maximizing the quality while minimizing the energy. The optimized drying time of 5.78 h for FD resulted in a QI of 0.68 and an SECI of 0.64. Compared to the drying time of 8 h, the QI and SECI improved by 9.7% and 30.6%. However, compared to a lower experimental drying time of 4 h, QI and SECI decreased slightly by 1.5% and 14%, respectively. This intermediate optimization is essential, as it falls between the experimental times of 4 h and 8 h, capturing the benefits of reduced energy consumption while maintaining high product quality across the drying time.

The optimized drying time of 3.81 h for ID yielded significant improvements in both product quality and energy efficiency. A QI of 0.47 and SECI of 0.94 represent an 11.9% increase in QI compared to the highest experimental QI, achieved at a reduced drying time of 4.8%. Additionally, the SECI improved by 1.1%. These results demonstrate the value of optimizing the drying time, enhancing product quality, and slightly improving energy efficiency, making the process more effective and sustainable.

An optimized drying time of 4.64 h for OD resulted in a QI of 0.44 and an SECI of 0.99. Compared to drying times of 4 h and 8 h, the optimized QI represents a 2.3% and 83.3% increase, respectively, with a slight decrease in SECI by 1% and 7.6%. This indicates that a slight increase in drying time can lead to better quality with a minimal compromise on energy efficiency. These results emphasize the importance of fine-tuning drying times to achieve an optimal balance between product quality and energy use, maximizing economic and environmental sustainability.

The optimization process also highlights the differing behaviour of the QI and SECI across the drying methods, as indicated in Figure 4c, the slope of the graph shows that the QI changes minimally with drying time for FD. This suggests that FD maintains high quality even at extended drying times. In contrast, OD shows a significant change in QI with drying time, indicating a higher sensitivity to drying duration. The SECI for OD changes minimally, suggesting stable energy consumption, while FD shows significant changes, indicating higher energy usage variability.

## 4. Conclusions

The study investigated the effect of three drying methods (FD, ID, and OD) based on the morphological features and the post-processing quality characteristics of potatoes. The results showed that the three tested drying methods significantly influenced the overall quality of dried potato samples. The designed HQSE could precisely describe the changes in product quality and specific energy consumption across the drying methods. It was found that FD performed substantially well compared to the other two tested drying methods, maintaining a better CQI across all drying times. However, FD had the lowest SECI, while ID and OD showed a significantly higher (*p* < 0.05) SECI, indicating better energy consumption. Implementing multi-objective optimization allowed for a detailed analysis of the trade-offs between optimal quality and energy consumption.

The developed mathematical models accurately described the quality and energy consumption changes during the drying process under three drying methods. The findings provide essential information for designing hybrid drying systems for potato cubes to leverage the strengths of different drying methods at various stages to maximize overall process efficiency. The study was limited to a specific potato variety and laboratory-scale drying methods. Future research should explore a broader range of food materials and scale up to industrial levels to optimize hybrid drying systems. Further studies should also focus on integrating these methods into a single hybrid dryer unit for better efficiency and quality control.

## Figures and Tables

**Table 1:** Developed regression models for different drying methods.

Drying Method	Linear Regression Formula	Coefficient of Determination (R^2^)	Polynomial Regression Formula	Coefficient of Determination (R^2^)
FD	SECI=−0.058t+0.9706	0.97	$QI=0.0012t^{2}-0.0305t+0.7921$	0.99
ID	SECI=−0.0352t+1.0767	0.99	$QI=0.0022t^{2}-0.059t+0.631$	0.91
OD	SECI=−0.0215t+1.0892	0.99	$QI=0.033t^{2}-0.087t+0.7302$	0.99

Note: SECI represents specific energy consumption index, *QI* represents quality index at, *t* drying time.

**Table 2:** Effect of different drying methods and drying duration on morphological features of potato samples.

Drying Method	Drying Time (h)	Close Porosity (%)	Open Porosity (%)	Area Shrinkage Ratio	Volume Shrinkage Ratio
FD	4	13.07 ± 0.15 ^ef^	3.43 ± 0.71 ^b^	0.50 ± 0.17 ^g^	0.67 ± 0.23 ^g^
	8	35.60 ± 0.04 ^c^	0.92 ± 0.18 ^bc^	1.75 ± 0.66 ^g^	2.32 ± 0.87 ^g^
	12	42.40 ± 0.06 ^b^	0.53 ± 0.17 ^bc^	5.00 ± 1.41 ^f^	7.14 ± 1.84 ^f^
	16	52.32 ± 0.05 ^a^	0.37 ± 1.16 ^bc^	12.15 ± 1.01 ^e^	18.50 ± 1.23 ^e^
IR	4	0.00 ± 0.00 ^h^	6.82 × 10^−4^ ± 0.0 ^d^	37.13 ± 0.48 ^c^	49.48 ± 0.52 ^c^
	8	3.73 ± 0.98 ^g^	4.22 ± 0.95 ^b^	39.34 ± 0.83 ^c^	52.35 ± 0.87 ^c^
	12	10.70 ± 0.18 ^f^	0.32 ± 1.21 ^d^	63.84 ± 1.89 ^a^	76.73 ± 1.72 ^a^
	16	15.00 ± 0.59 ^de^	10.50 ± 0.70 ^a^	64.34 ± 0.85 ^a^	77.25 ± 0.77 ^a^
OD	4	0.00 ± 0.00 ^h^	6.3 × 10^−4^ ± 0.00 ^d^	31.55 ± 0.76 ^d^	42.31 ± 0.88 ^d^
	8	5.45 ± 0.23 ^g^	0.31 ± 0.26 ^bc^	50.97 ± 0.41 ^b^	63.91 ± 0.43 ^b^
	12	6.49 ± 0.38 ^g^	1.23 ± 1.22 ^c^	65.20 ± 1.74 ^a^	78.22 ± 1.55 ^a^
	16	16.80 ± 0.49 ^d^	2.65 ± 0.55 ^b^	65.98 ± 0.18 ^a^	79.05 ± 0.16 ^a^

Data are expressed as mean ± SD values; significant difference (*p* < 0.05) within a column for each treatment combination (drying method and time) is denoted by lowercased letters. FD refers to “Freeze Drying”, ID—“Infrared Drying” and OD—“Oven Drying”.

**Table 3:** Effect of different drying methods and drying times on colour, hardness, and density changes in potato samples.

Drying Method	Drying Time (h)	L*	a*	b*	∆E	Hardness (N)	Density (kgm^−3^)
FD	4	98.48 ± 0.50 ^a^	−2.43 ± 0.38 ^c^	10.62 ± 0.89 ^d^	4.79 ± 0.20 ^d^	17.12 ± 7.96 ^e^	−52.99 ± 0.82 ^e^
	8	98.21 ± 0.69 ^ab^	−1.71 ± 0.44 ^bc^	10.04 ± 0.54 ^d^	5.33 ± 0.60 ^cd^	24.16 ± 4.43d ^e^	−67.03 ± 0.11 ^f^
	12	97.30 ± 0.88 ^abc^	−1.20 ± 0.13 ^abc^	10.78 ± 0.61 ^cd^	5.71 ± 0.15 ^bcd^	35.03 ± 6.26 ^de^	−71.41 ± 1.94 ^f^
	16	96.47 ± 1.22 ^abcd^	−1.26 ± 0.20 ^abc^	11.73 ± 0.41 ^bcd^	5.82 ± 0.12 ^bcd^	18.00 ± 3.32 ^e^	−72.09 ± 0.41 ^f^
ID	4	96.20 ± 0.69 ^abcde^	−1.95 ± 0.52 ^c^	13.40 ± 0.64 ^abc^	5.80 ± 0.15 ^bcd^	39.89 ± 8.27d ^e^	−21.61 ± 0.34 ^d^
	8	95.72 ± 0.72 ^bcde^	−1.27 ± 0.27 ^abc^	13.95 ± 0.81 ^ab^	6.19 ± 0.69 ^bcd^	72.26 ± 18.29 ^c^	−46.57 ± 0.47 ^e^
	12	93.96 ± 0.54 ^def^	−0.69 ± 0.42 ^ab^	14.63 ± 0.50 ^a^	6.30 ± 0.43 ^bc^	115.78 ± 6.67 ^b^	−6.58 ± 6.86 ^c^
	16	92.50 ± 1.43 ^fg^	−0.70 ± 0.46 ^ab^	15.53 ± 0.61 ^a^	6.65 ± 0.60 ^b^	184.68 ± 9.15 ^a^	−9.21 ± 4.64 ^c^
OD	4	95.47 ± 1.19 ^cde^	−1.59 ± 0.31 ^abc^	13.86 ± 0.50 ^ab^	5.97 ± 0.18 ^bcd^	23.85 ± 5.97 ^de^	−24.14 ± 1.28 ^d^
	8	93.85 ± 0.46 ^ef^	−1.25 ± 0.21 ^abc^	14.41 ± 0.45 ^a^	6.32 ± 0.12 ^bc^	45.82 ± 11.97 ^d^	−20.10 ± 0.66 ^d^
	12	91.88 ± 0.73 ^fg^	−1.44 ± 0.35 ^abc^	14.67 ± 0.98 ^a^	6.81 ± 0.11 ^ab^	110.74 ± 6.75 ^b^	12.54 ± 2.27 ^a^
	16	91.24 ± 0.89 ^g^	−0.56 ± 0.26 ^a^	14.63 ± 2.04 ^a^	7.99 ± 0.08 ^a^	165.25 ± 8.08 ^a^	3.79 ± 0.52 ^b^

Data are expressed as mean ± SD values; significant difference (*p* < 0.05) within a column for each treatment combination (drying method and time) is denoted by lowercased letters. FD refers to “Freeze Drying”, ID—“Infrared Drying” and OD—“Oven Drying”. L*—Lightness, a*—hue between red and green, b*—hue between yellow and blue, and ΔE—the degree of difference in colour.

## Data Availability

The original contributions presented in the study are included in the article; further inquiries can be directed to the corresponding author.
